# Monocentric evaluation of Ki-67 labeling index in combination with a modified RPA score as a prognostic factor for survival in IDH-wildtype glioblastoma patients treated with radiochemotherapy

**DOI:** 10.1007/s00066-022-01959-6

**Published:** 2022-05-25

**Authors:** R. Dumke, C. Dumke, F. Eberle, Ch. Nimsky, U. Keber, R. Engenhart-Cabillic, S. Lautenschläger

**Affiliations:** 1 Department of Radiation Oncology, Marburg University Hospital, Marburg, Hessen Germany; 2 Marburg Ion-Beam Therapy Center (MIT), Department of Radiation Oncology, Marburg University Hospital, Marburg, Hessen Germany; 3 Vivantes Radiotherapy, MVZ Spandau, Berlin, Germany; 4 Department of Neurosurgery, Marburg University Hospital, Marburg, Hessen Germany; 5 Institute of Neuropathology, Marburg University Hospital, Marburg, Hessen Germany; 6 Department of Radiotherapy, Lippe Hospital, Lemgo, Nordrhein-Westfalen Germany

**Keywords:** Recurrence, MGMT, ATRX, p53, EGFR

## Abstract

**Purpose:**

The prognosis for glioblastoma patients remains dismal despite intensive research on better treatment options. Molecular and immunohistochemical markers are increasingly being investigated as understanding of their role in disease progression grows. O(6)-methylguanine-DNA methyltransferase (MGMT) promoter methylation has been shown to have prognostic and therapeutic relevance for glioblastoma patients. Other markers implicated in tumor formation and/or malignancy are p53, Alpha thalassemia/mental retardation syndrome X-linked (ATRX), Epidermal Growth Factor Receptor splice variant III (EGFRvIII), and Ki-67, with loss of nuclear ATRX expression and lower Ki-67 index being associated with prolonged survival. For p53 and EGFRvIII the data are contradictory. Our aim was to investigate the markers mentioned above regarding progression-free (PFS) and overall survival (OS) to evaluate their viability as independent prognostic markers for our patient collective.

**Methods:**

In this retrospective study, we collected data on patients undergoing radiotherapy due to isocitrate dehydrogenase (IDH) wildtype glioblastoma at a single university hospital between 2014 and 2020.

**Results:**

Our findings confirm Ki-67 labeling index ≤ 20% as an independent prognostic factor for prolonged PFS as well as MGMT promoter methylation for both prolonged PFS and OS, in consideration of age and Eastern Cooperative Oncology Group (ECOG) status, chemotherapy treatment, and total radiation dose for PFS as well as additionally sex, resection status, and receipt of treatment for progression or recurrence for OS. Additionally, Ki-67 labeling index ≤ 20% showed a significant correlation with prolonged OS in univariate analysis. Modification of the recursive partitioning analysis (RPA) score to include Ki-67 labeling index resulted in a classification with the possible ability to distinguish long-term-survivors from patients with unfavorable prognosis.

**Conclusion:**

MGMT promoter methylation and Ki-67 labeling index were independent predictors of survival in our collective. We see further studies pooling patient collectives to reach larger patient numbers concerning Ki-67 labeling index as being warranted.

**Supplementary Information:**

The online version of this article (10.1007/s00066-022-01959-6) contains supplementary material, which is available to authorized users.

## Introduction

Glioblastomas (GBM) account for 15–20% of brain tumors [1–4] and have a dismal prognosis with median overall survival (OS) ranging from about 5 to 15 months [3–6]. Standard treatment consists of maximum safe resection followed by radio-, chemo-, or radiochemotherapy according to patient characteristics [7]. Younger patients with good performance score (PS) of Eastern Cooperative Oncology Group status (ECOG) 0–2 receive 60 Gy radiotherapy with concomitant and adjuvant temozolomide (TMZ) chemotherapy according to the Stupp protocol [8]. Elderly patients with good PS receive hypofractionated radiotherapy in case of unmethylated O(6)-methylguanine-DNA methyltransferase (MGMT) promoter or temozolomide (TMZ) chemotherapy in case of methylated MGMT promoter according to the NORDIC trial [9]. For elderly patients with an unfavorable PS, best supportive care is recommended [7]. In case of recurrence, an interdisciplinary tumor board evaluates options for renewed resection, reirradiation, and/or salvage chemotherapy [7].

Efforts have been made to find reliable prognostic markers for survival, with increased focus turning to protein expression and molecular markers due to mounting evidence of “genotype trump[ing] the histological phenotype” [10].

P53 is a transcription factor which functions as a tumor suppressor via regulation of cell cycle control, apoptosis, cell differentiation, and neovascularization [11–13], and loss of normal p53 function, e.g., via mutation of its encoding gene, *TP53*, plays an early role in tumor formation [14, 15]. *TP53* mutations occur mainly, but not exclusively, in secondary GBM, isocitrate dehydrogenase (IDH) mutant [12, 13, 16, 17], and can contribute to chemotherapy resistance [18, 19]. To date, no conclusive argument can be made for a clear correlation of *TP53* mutation and survival, with several studies finding no association [14, 18, 20–24] whereas some studies showed a survival benefit [13, 25].

Alpha thalassemia/mental retardation syndrome X-linked (ATRX) plays a role in genomic stability [26] and regulation of cell division [27]. *ATRX* mutations cause genomic instability, non-homologous end-joining (NHEJ), and alternate lengthening of telomeres (ALT) [28–32], as well as increasing susceptibility to DNA-damaging chemotherapy [29]. Loss of nuclear ATRX expression due to *ATRX* gene mutation [31] has been correlated with a survival benefit [33, 34] and is mainly seen in secondary GBM, IDH mutant [35, 36].

Epidermal growth factor receptor (EGFR) plays a central role in cell proliferation, differentiation [37–39], cell cycle [40], and angiogenesis [41], as well as influencing DNA repair and contributing to radio- and chemotherapy resistance [42]. Amplification of the *EGFR* gene occurs in 30–50% of IDH-wildtype GBMs [5, 13, 16, 43–49] and is correlated with overexpression of EGFR protein [12, 46, 47, 49]. The most common mutated variant in GBM is tumor-specific EGFR splice variant III (EGFRvIII) which results from gene truncation [16, 50–52] and has been associated with worse survival prognosis [53] and increased carcinogenicity [54]. Current data concerning the survival correlation of EGFR are inconclusive, with some studies showing shorter survival [43, 44, 48, 55] and some studies showing no correlation [18, 24, 25, 53, 56] with survival. A large meta-analysis concluded that most studies did not regard confounders and/or did not differentiate *EGFR* wildtype from *EGFR* mutations and so no clear statement could be made regarding* EGFR* status and survival prognosis [57].

Ki-67 is expressed exclusively in proliferating cells [58, 59], with the Ki-67 labeling index (Ki-67 LI; % of cells expressing Ki-67) correlating closely with histological tumor grade in gliomas [14, 60–64] and a Ki-67 LI of 10% being regarded as a reliable criterium for malignancy [65, 66]. Although some studies see Ki-67 LI as a reliable predictor of survival with higher Ki-67 LI indicating worse prognosis [14, 58, 66–70], more reliable even than histologic grade [44, 71] or age [44], other studies could not find this association [56, 72–74], possibly due to low reproducibility of Ki-67 LI detection between laboratories and examiners [75]. Therefore, the role of Ki-67 LI as a prognostic factor is still disputed.

O(6)-methylguanine-DNA methyltransferase (MGMT) is involved in repair of DNA strands by removing genotoxic alkyl groups from guanine [76, 77]. Methylation of the MGMT promoter reduces MGMT expression and thereby increases sensitivity to alkylating chemotherapy [78–81]. Therefore, MGMT promoter methylation is consistently correlated with better survival when alkylating treatment has been performed [24, 82–86].

Our aim was to investigate the markers mentioned above regarding progression-free (PFS) and overall survival (OS) to evaluate their viability as independent prognostic markers for our patient collective.

## Materials and methods

This study was a single-center retrospective cohort study conducted at the Department of Radiation Oncology of the University Hospital Marburg. Inclusion criterium for the study was receipt of radiation therapy due to an IDH-wildtype glioblastoma during 2014 to 2020. For these patients, data regarding patient, disease, and treatment characteristics as well as immunohistochemical and molecular marker status were collected from archived files. Subsequently, progression-free and overall survival was calculated, with progression-free survival being defined as time from first diagnosis to first progression or relapse in magnetic resonance imaging (MRI) or death and overall survival being defined as time from first diagnosis to death or last follow up.

In our clinic, standard treatment for patients closely follows guidelines, with patients undergoing maximum safe resection with 5-aminolevulinic acid fluorescence image-guided surgical resection (5-ALA-FIGR) or biopsy when maximum resection is not feasible and subsequent radio-, chemo-, or radiochemotherapy. Target delineation mostly follows ESTRO-ACROP (European Society for Radiation and Oncology - Advisory Committee on Radiation Oncology Practice) guidelines [87]. Firstly, gross tumor volume (GTV) is defined as encompassing the resection cavity or residual enhancing tumor in contrast-enhanced T1-weighted MRI on the one hand (GTV tumor) and as encompassing edema in T2-weighted MRI on the other (GTV edema). Clinical target volume (CTV) is then delineated by adding a safety margin of 1.5 cm to the GTV tumor and matching this volume with GTV edema. Lastly, the planning target volume (PTV) adjusts the CTV for anatomical features and organs at risk as well as adding 0.5 cm to account for possible imprecision during patient positioning. Radiation techniques used are 3D conformal external beam radiotherapy and intensity-modulated radiotherapy (IMRT) and use mainly photon beams with proton beams being added as an optional 10 Gy boost. Younger patients with good PS receive radiochemotherapy according to the Stupp protocol [8], consisting of 60 Gy total dose either as 60 Gy of photons or 50 Gy of photons plus a 10 Gy proton boost with concomitant (75 mg/m^2^) and adjuvant (1 course 150 mg/m^2^ day 1–5 + 5 courses 200 mg/m^2^ day 1–5) TMZ. Since 2019, these patients have also received lomustine (CCNU; 6 courses 100 mg/m^2^ lomustine day 1 + 100 mg/m^2^ TMZ day 2–6) according to the CeTeG/NOA-09 protocol [88] when the MGMT promoter is methylated. According to the NORDIC/NOA-08 trial [9], elderly patients receive 34 to 40.5 Gy total-dose radiotherapy when the MGMT promoter is unmethylated and either the same or TMZ alone when the MGMT promoter is methylated. In case of recurrence, further therapy is discussed in an interdisciplinary tumor board and patients receive either renewed resection whenever feasible and/or reirradiation either with carbon ions (CIRT) or fractionated stereotactic radiotherapy (FSRT) with photons and/or salvage chemotherapy either with TMZ (as a prolonged or dose-intense course) or lomustine [89].

Protein expression was determined by neuropathological evaluation of biopsy or resection tissue. Immunohistochemistry was performed as described previously [90]. In brief, heat-induced epitope retrieval was performed with either citrate or ethylenediaminetetraacetic acid (EDTA) according to the manufacturer’s protocol of the respective primary antibody. Sections were incubated for 1 hour with the following primary antibodies: anti-p53 (1:100; Dako M7001, Agilent Technologies, Inc., Santa Clara, CA, USA), anti-ATRX (1:150; Sigma HPA 001906, Sigma-Aldrich Chemie GmbH, Taufkirchen, Germany), anti-EGFRvIII (1:50; Zytomed MSK029-05, Zytomed Systems GmbH, Berlin, Germany), and anti-Ki-67/MiB‑1 (1:200; Dako M7240, Agilent Technologies, Inc., Santa Clara, CA, USA). Sections were washed and incubated with post-block solution and horse radish peroxidase (HRP) polymer reagent according to the manufacturer’s protocol of the ZytoChem-Plus HRP Polymer Kit (Zytomed Systems GmbH, Berlin, Germany). According to the World Health Organisation (WHO) classification of tumors of the central nervous system (2021, [91]) a strong nuclear expression of p53 in more than 10% of tumor cells and cytoplasmic EGFRvIII expression of either partial or entire tumor were defined as positive, respectively. Nuclear ATRX loss was diagnosed in case of negative tumor cells among positive endothelial cells serving as an internal control. Ki-67 LI demonstrates the percentage of immunoreactive tumor cells from all tumor cells.

MGMT promoter methylation status was examined by methylation-specific polymerase chain reaction (MSP) as described previously [92, 93]. In brief, DNA was isolated from paraffin sections of the tumor using the DNeasy Blood and Tissue Kit (QUIAGEN GmbH, Hilden, Germany). A total of 500 ng DNA was treated with sodium bisulfite using the EZ DNA Methylation Gold Kit (Zymo Research Corp., Irvine, CA, USA). The primer sequences used to detect unmethylated MGMT promoter sequences were 5‑TGT GTT TTT AGA ATG TTT TGT GTT TTG AT‑3 and 5‑CTA CCA CCA TCC CAA AAA AAA ACT CCA‑3. The primer sequences used to detect methylated MGMT promoter sequences were 5‑GTT TTT AGA ACG TTT TGC GTT TCG AC‑3 and 5‑CAC CGT CCC GAA AAA AAA CTC CG‑3.

For data collection and analysis, we used IBM® SPSS® Statistics (version 21; IBM Corp., Armonk, NY, USA). The prevalence of investigated variables as well as the calculation of means and standard deviations was obtained by descriptive statistics. Kaplan–Meier survival analysis was used to determine progression-free and overall survival. All tests with *p* < 0.05 were then included in univariate analysis (log-rank test) for comparison of survival probability. Following this, all tests with *p* < 0.1 were included in multivariate analysis using a Cox proportional hazards model to analyze possible dependencies. Lastly, tests with *p* < 0.05 in multivariate analysis were considered significant. Wilcoxon signed-rank test was used for analysis of Ki-67 LI change upon recurrence.

The study was inspected and approved by the Ethics committee of the Philipps-Universität Marburg (ethics vote “Studie 166/18”).

## Results

Data were collected from patients treated between 2014 and 2020. Median follow-up was 13.4 months. A total of 137 patients were included in the study, with 59.9% being male (*n* = 82/137) and 40.1% being female (*n* = 55/137). The median age at diagnosis was 63.0 years (24.9–84.6). ECOG status at diagnosis could be determined for 87.6% of patients, with most (82.5%, *n* = 113/137) ranging from 0 to 2 (Table 1). Tumor localization was diverse (Table 1), with complete or partial resection achievable for 84.6% of patients (42.3%, *n* = 58/137, each). 71.5% (*n* = 98/137) of patients suffered a progression or recurrence and 74.5% (*n* = 102/137) had died by the end of the data collection period. The majority of patients underwent chemotherapy, with 81.0% (*n* = 111/137) receiving either temozolomide (TMZ) alone (71.5%, *n* = 98/137) or TMZ plus lomustine (CCNU; 9.5%, *n* = 13/137). Most patients showed ATRX (91.2%, *n* = 125/137) and p53 expression (84.7%, *n* = 116/137), whilst EGFRvIII expression (40.9%, *n* = 56/137) was less common. The MGMT promoter was methylated in 61.3% (*n* = 84/137) of patients. Ki-67 LI averaged 20% (0–80%). 72.2% (*n* = 99/137) of patients received a total radiation dose of 60 Gy, either as sole photon irradiation (45.5%, *n* = 45/99) or as 50 Gy photon irradiation plus a 10-Gy proton boost (54.5%, *n* = 54/99).

**Table 1:** Patient characteristics in the overall collective

	*n* (%)
**Total**	137 (100)
**Sex**	
*Male*	82 (59.9)
*Female*	55 (40.1)
**ECOG at primary diagnosis**	
*0*	50 (36.5)
*1*	44 (32.1)
*2*	19 (13.9)
*3–4*	7 (5.1)
*Unknown*	17 (12.4)
**Location of primary tumor**	
*Frontal lobe*	32 (23.4)
*Temporal lobe*	32 (23.4)
*Parietal lobe*	17 (12.4)
*Other*	16 (11.7)
*>* *1 area*	40 (29.2)
**Marker status**	
*ATRX expression*	
Yes	125 (91.2)
No	6 (4.4)
Unknown	6 (4.4)
*EGFRvIII overexpression*	
Yes	56 (40.9)
No	74 (54.0)
Unknown	7 (5.1)
*p53 overexpression*	
Yes	116 (84.7)
No	10 (7.3)
Unknown	11 (8.0)
*MGMT promoter*	
Non-methylated	52 (38.0)
Methylated	84 (61.3)
Unknown	1 (0.7)
*Ki-67 LI*	
≤ 20%	82 (59.9)
> 20%	50 (36.5)
Unknown	5 (3.6)

*ECOG* Eastern Cooperative Oncology Group status, *ATRX* Alpha thalassemia/mental retardation syndrome X-linked, *EGFRvIII* Epidermal Growth Factor Receptor splice variant III, *MGMT* O(6)-methylguanine-DNA methyltransferase, *Ki-67 LI* Ki-67 labeling index

Details of first and second progression or recurrence as well as retreatment modalities are listed in Table 2. Median time to beginning of retreatment after diagnosis of progression or recurrence was 1.5 weeks. Re-reirradiation was administered only in case of out-of-field recurrence of first reirradiation.

**Table 2:** Treatment characteristics in overall collective

	*n* (%)
**Resection status**	
*Biopsy*	18 (13.1)
*Partial resection*	58 (42.3)
*Complete resection*	58 (42.3)
**Chemotherapy (TMZ/TMZ** **+** **CCNU)**	
*Yes*	111 (81.0)
*No*	23 (16.8)
**Radiotherapy**	
*60* *Gy*	45 (32.8)
*50* *Gy photons* *+* *10* *Gy protons*	54 (39.4)
*40.5* *Gy*	21 (15.3)
*34* *Gy*	7 (5.1)
*Other*	10 (13.7)
**Status**	
*Alive*	35 (25.5)
*Dead*	102 (74.5)
**Progression or recurrence**	
*Yes*	98 (71.5)
*No*	39 (28.5)
**Treatment of progression or recurrence**	
*Resection only*	9 (9.2)
*Radiotherapy only*	23 (23.5)
*Chemotherapy only*	5 (5.1)
*Combination of two of the above*	26 (26.5)
Resection and radiotherapy	12 (12.2)
Resection and chemotherapy	9 (9.2)
Radio- and chemotherapy	4 (4.1)
*Combination of three of the above*	11 (11.2)
*None*	24 (24.5)
**Re-progression or re-recurrence**	
*Yes*	26 (26.5)
*No*	72 (73.5)
**Treatment of re-progression or re-recurrence**	
*Resection only*	1 (3.8)
*Radiotherapy only*	7 (26.9)
*Chemotherapy only*	4 (15.4)
*Combination of two of the above*	4 (15.4)
Resection and radiotherapy	2 (7.7)
Resection and chemotherapy	2 (7.7)
*None*	10 (38.5)

*TMZ* temozolomide, *CCNU* lomustine

**Fig. 1:**
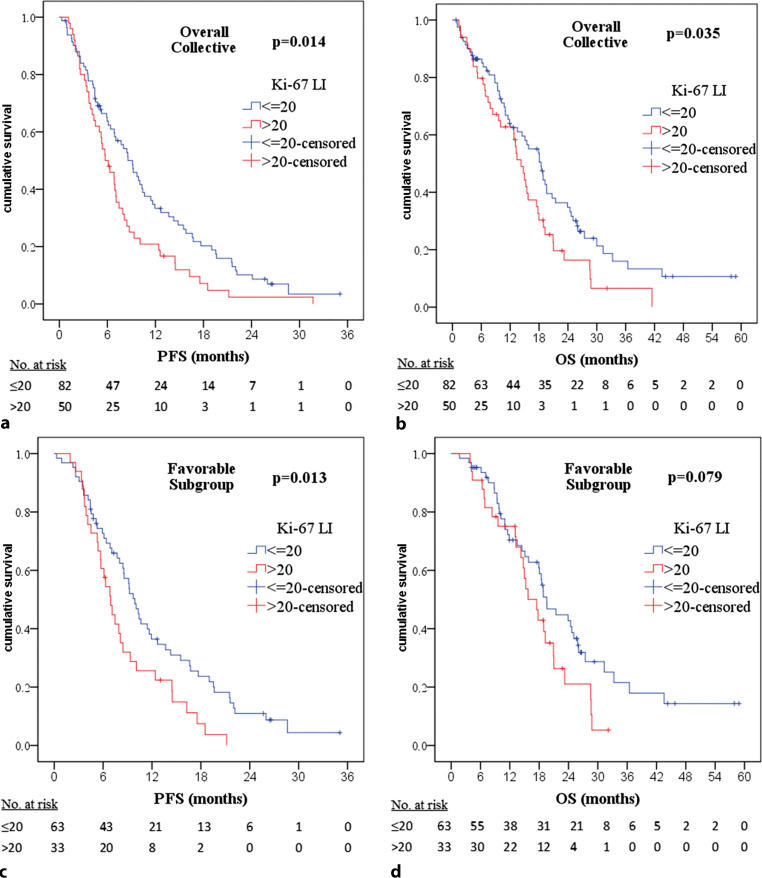
Kaplan–Meier plots and *p*-values of Kaplan–Meier survival analysis for progression-free survival (*PFS*) and overall survival (*OS*) regarding Ki-67 labeling index (*Ki-67 LI*). For the overall collective: **a** PFS significant with *p* = 0.014. **b** OS significant with *p* = 0.035. For partial collective “favorable subgroup”: **c** PFS significant with *p* = 0.013. **d** OS not significant with *p* = 0.079

**Fig. 2:**
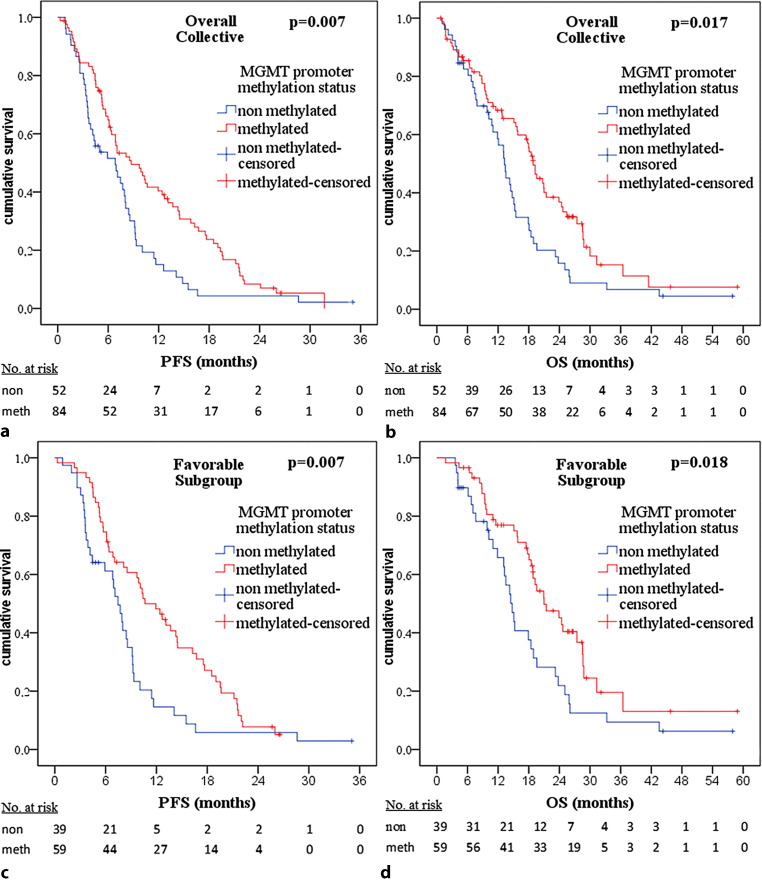
Kaplan–Meier plots and *p*-values of Kaplan–Meier survival analysis for progression-free survival (*PFS*) and overall survival (*OS*) regarding O(6)-methylguanine-DNA methyltransferase (*MGMT*) promoter methylation status. For overall collective: **a** PFS significant with *p* = 0.007. **b** OS significant with *p* = 0.017. For partial collective “favorable subgroup”: **c** PFS significant with *p* = 0.007. **d** OS significant with *p* = 0.018

For the overall collective, median progression-free survival (PFS) was 7.5 (6.3–8.6) months. Regarding Ki-67 LI in the overall collective, median PFS was 8.6 (6.6–10.6) months for patients with an index of ≤ 20% compared to 5.7 (4.0–7.5) months in case of > 20% (*p* = 0.014; Fig. 1a). For MGMT promoter methylation status, PFS in the overall collective was 6.8 (3.6–10.1) months for patients with a non-methylated MGMT promoter compared to 8.7 (5.5–11.9) months in case of a methylated MGMT promoter (*p* = 0.007, Fig. 2a).

Overall survival was median 15.7 (13.3–18.2) months for the overall collective. Regarding Ki-67 LI, OS in the overall collective was median 18.4 (15.2–21.7) months for patients with an index of ≤ 20% compared with 14.3 (11.9–16.6) months in case of > 20% (*p* = 0.035, Fig. 1b). For MGMT promoter methylation status, median OS in the overall collective was 13.2 (10.9–15.5) months for patients with a non-methylated MGMT promoter compared to 18.8 (17.2–20.6) months in case of a methylated MGMT promoter (*p* = 0.017, Fig. 2b).

For those patients receiving a total radiation dose of 60 Gy (72.2%, *n* = 99/137), we performed a subgroup analysis (partial collective “favorable subgroup,” FS) which showed better ECOG status and lower age (60.6%, *n* = 60/99, below median age) than in the overall collective as well as similar stats for sex, resection status, and rate of progression or recurrence as the overall collective (Supplement Table S1 and Supplement Table S2). Relatively, more patients received chemotherapy than in the overall collective (89.9%, *n* = 89/99) and by the end of data collection, fewer patients had died (68.7%, *n* = 68/99). Again, most patients showed ATRX (88.9%, *n* = 88/99) and p53 expression (83.8%, *n* = 83/99), with EGFRvIII expression (50.5%, *n* = 50/99) more common than in the overall collective. The MGMT promoter was methylated in 59.6% (*n* = 59/99) of patients and 63.6% (*n* = 63/99) of patients showed equal to or less than 20% Ki-67 labeling index.

For this favorable subgroup, median PFS was 9.1 (7.6–10.7) months. Regarding Ki-67 LI, PFS was 9.9 (8.5–11.2) months for patients with an index of ≤ 20% compared to 6.9 (5.6–8.3) months in case of > 20% (*p* = 0.013, Fig. 1c). Concerning MGMT promoter methylation, PFS was 7.5 (6.1–8.8) months for patients with a non-methylated MGMT promoter compared to 10.6 (7.5–13.8) months in case of a methylated MGMT promoter (*p* = 0.007, Fig. 2c).

Overall survival was median 18.9 (17.2–20.5) months for the favorable subgroup. Regarding Ki-67 LI, no statistically significant difference in OS could be found (*p* = 0.079, Fig. 1d). Concerning MGMT promoter methylation, median OS was 14.8 (1.6–16.9) months for patients with a non-methylated MGMT promoter compared to 21.0 (15.7–26.3) months in case of a methylated MGMT promoter (*p* = 0.018, Fig. 2d).

In case of ATRX and p53 expression, no statistically significant differences in PFS or OS could be found for either the overall collective or for the favorable subgroup (data not shown). EGFRvIII expression was only borderline significant for the overall collective (*p* = 0.050, data not shown).

**Fig. 3:**
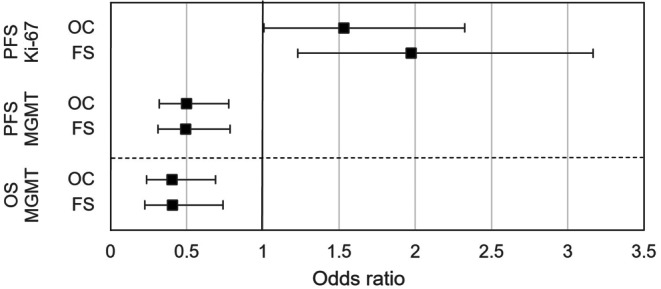
Forest plot for odds ratios and 95% confidence intervals from multivariate analysis regarding progression-free survival (*PFS*) and overall survival (*OS*) for Ki-67 labeling index (*Ki-67*) and O(6)-methylguanine-DNA methyltransferase promoter methylation status (*MGMT*) for the overall collective (*OC*) and the partial collective “favorable subgroup” (*FS*)

Multivariate analysis using a Cox proportional hazards model was conducted for the overall collective and repeated for the favorable subgroup, and included the variables listed in Table 3. Regarding Ki-67 LI, increased odds ratios for an index > 20% were statistically significant for the overall collective and the favorable subgroup regarding PFS but not for OS. For MGMT promoter methylation status, a decrease in odds ratio for a methylated MGMT promoter was statistically significant for both the overall collective as well as for the favorable subgroup regarding PFS and OS. Details concerning odds ratios can be found in Fig. 3.

**Table 3:** Variables included in multivariate analysis with a Cox proportional hazards model

Collective	Survival	Variables
Overall	PFS	ECOG at diagnosis EGFRvIII expression MGMT promoter methylation status* Ki-67 LI* Chemotherapy treatment Total radiation dose*
	OS	Age at diagnosis* ECOG at diagnosis Sex Resection status* MGMT promoter methylation status* Ki-67 LI Chemotherapy treatment Total radiation dose Receival of re-treatment*
FS	PFS	MGMT promoter methylation status* Ki-67 LI*
	OS	Age at diagnosis ECOG at diagnosis Resection status* MGMT promoter methylation status* Ki-67 LI Receipt of retreatment*

Variables included had reached *p* ≤ 0.1 in univariate analysis (Log-Rank Test)

*Variables marked with * reached *p* < 0.05 in multivariate analysis, *ECOG* Eastern Cooperative Oncology Group status, *ATRX* Alpha thalassemia/mental retardation syndrome X-linked, *EGFRvIII* Epidermal Growth Factor Receptor splice variant III, *MGMT* O(6)-methylguanine-DNA methyltransferase, *Ki-67 LI* Ki-67 labeling index

**Fig. 4:**
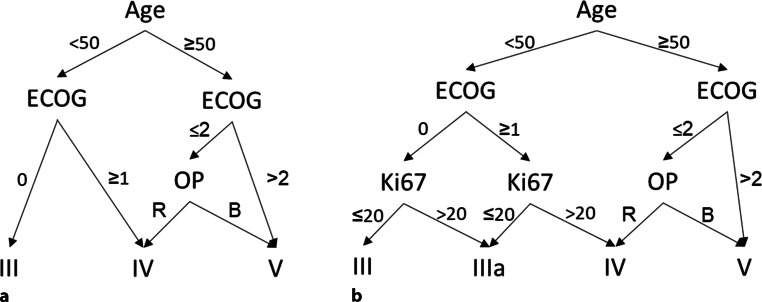
Decision trees for modified Radiation Therapy Oncology Group/European Organistion for Research and Treatment of Cancer (RTOG/EORTC) recursive partitioning analysis (RPA) score classes. **a** Modification of the simplified RTOG RPA classification from 2011 [94] excluding work status and including age, Eastern Cooperative Oncology Group status (*ECOG*), and resection status (*OP*, *R* resection, *B* biopsy): RPAmod. **b** Further modification of decision tree RPAmod to further include Ki-67 labeling index (*Ki67*)I: RPAki. *Age* in years, *Ki67* in %

**Fig. 5:**
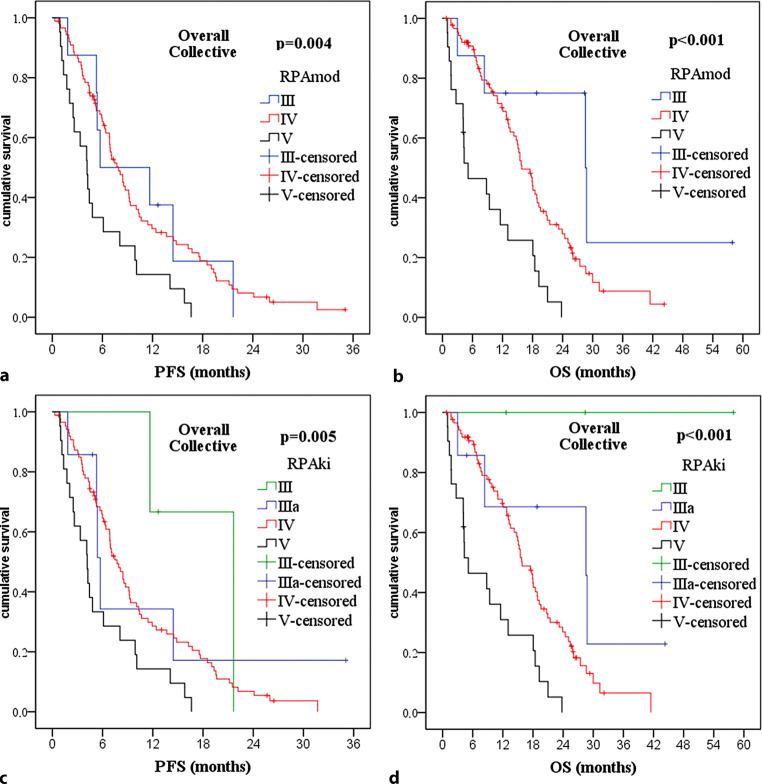
Kaplan–Meier-plots and *p*-values of Kaplan–Meier survival analysis for progression-free survival (*PFS*) and overall survival (*OS*) regarding modified recursive partitioning analysis (*RPAmod*) scores in the overall collective. For RPAmod: **a** PFS significant with *p* = 0.004. **b** OS significant with *p* < 0.001. For RPAki: **c** PFS significant with *p* = 0.005. **d** OS significant with *p* < 0.001

Due to the encouraging results regarding Ki-67 LI, we considered possibilities for integrating Ki-67 LI into a prognostic score. To this end, we first aimed to test the existing simplified Radiation Therapy Oncology Group/European Organistion for Research and Treatment of Cancer (RTOG/EORTC) recursive partitioning analysis (RPA) score [94] in our collective. Because we did not collect data on the ability of patients to work, we simplified the score further to exclude this factor (“RPAmod,” Fig. 4a). Kaplan–Meier survival analysis comparing the resulting scoring classes III, IV, and V yielded significant *p*-values for both PFS (*p* = 0.004) and OS (*p* < 0.001) in the overall collective (Fig. 5) as well as a significant *p*-value for OS (*p* = 0.004) in the favorable subgroup (data not shown), with significance for PFS being missed (*p* = 0.097). Following this analysis, we modified our decision tree further to include Ki-67 LI for patients under 50 years of age, resulting in formation of a new class IIIa (“RPAki,” Fig. 4b). This improved *p*-values of Kaplan–Meier survival analysis, resulting in even more significant *p*-values for OS in the overall collective (*p* < 0.001, Fig. 5) and in the favorable subgroup (*p* = 0.003, data not shown). *P*-values for PFS changed only slightly (overall collective *p* = 0.005, favorable subgroup *p* = 0.090). Neither RPAmod nor RPAki could find a significant difference in survival between scoring classes III and IV (RPAmod) or IIIa and IV (RPAki) per se (RPA mod: PFS *p* = 0.925/0.704, OS *p* = 0.075/0.054; RPAki PFS *p* = 0.359/0.365, OS *p* = 0.133/0.104; for overall collective/favorable subgroup respectively). Furthermore, for RPAki, score class III contained only 3 patients with an unusually long PFS (median 12.7 months) and OS (median 28.4 months), which substantially surpassed median survival times of any other scoring class.

**Fig. 6:**
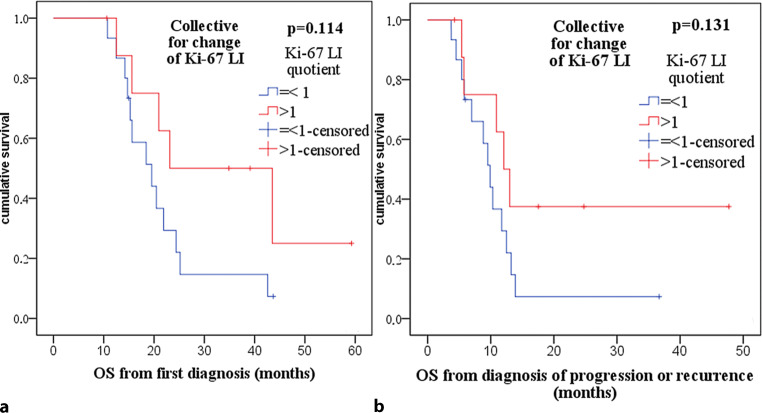
Kaplan–Meier-plots and *p*-values of Kaplan–Meier survival analysis regarding change in Ki-67 labeling index (*Ki-67 LI*) upon progression or recurrence (Ki-67 LI quotient = KI-67 LI upon initial diagnosis/Ki-67 LI upon progression or recurrence). **a** Overall survival (*OS*) from first diagnosis not significant with *p* = 0.114. **b** OS from diagnosis of progression or recurrence not significant with *p* = 0.131

To better understand the impact of (radio)therapy on Ki-67 LI, 24 patients were identified for whom Ki-67 LI upon recurrence or progression had been documented. Most of these patients (91.7%, *n* = 22/24) had received 60 Gy of photon irradiation. The quotient of initial Ki-67 LI and Ki-67 LI upon recurrence or progression showed statistical significance in the Wilcoxon signed-rank test for reduction of Ki-67 LI upon recurrence or progression (median quotient 2, *p* = 0.003), while the absolute median reduction of 6% was not statistically significant (*p* = 0.102). In Kaplan–Meier survival analysis, patients with a reduction of Ki-67 LI upon progression or recurrence (Ki-67 LI quotient > 1) showed a longer OS of 23.1 (17.4–24.5) months compared to 19.5 (12.6–26.5) months for patients with a stable or increased Ki-67 LI (Ki-67 LI quotient ≤ 1), although this difference did not reach statistical significance (*p* = 0.114, Fig. 6). Regarding overall survival from time of diagnosis of progression or recurrence, patients with a reduction of Ki-67 LI upon progression or recurrence (Ki-67 LI quotient > 1) again showed longer OS of 12.1 (9.1–15.0) months compared to 9.9 (8.0–11.8) months for patients with a stable or increased Ki-67 LI (Ki-67 LI quotient ≤ 1), although this difference did also not reach statistical significance (*p* = 0.131, Fig. 6).

## Discussion

Overall, our collective can be seen as representative of GBM patients, as median age of 63.0 years and overall survival of 15.7 months correspond to known data [2–6]. The slightly longer overall survival of 18.9 months in the favorable subgroup partial collective might be due to slightly better ECOG status in this collective, as a better performance status has been consistently correlated with improved survival [56, 67, 71, 74].

Expression of p53 was slightly higher than expected, with 84.7% of patients showing overexpression compared with 23 to 69% reported in the literature [17, 95–97]. Notably, in analyses, p53 protein overexpression is often equated with *TP53* gene mutations, since the degradation of mutated p53 protein is disturbed and p53 subsequently accumulates [98–100]. However, wildtype p53 can also be upregulated by diverse cellular stress signals [100, 101] which might account for the discrepancy from the lower percentages reported for genetically analyzed *TP53* mutations (25 to 54%, [12, 13, 16, 101–103]) [104]. Furthermore, the intensity of immunohistochemical staining can vary in dependence of the antibody and staining method used and the evaluation of overexpression is subject to the assessment of the examiner (being, by nature, not a purely quantitative method). This approach could lead to higher numbers of cases being classified as overexpressed compared to cut-offs used in other studies (e.g., [105]). In line with previous studies [14, 18, 20–24], we found no correlation between p53 expression and survival.

Loss of nuclear ATRX expression in 8.8% of our patients coincides with other studies (7–12% [35, 36]), with preservation of ATRX adding to malignancy as it is implicated in DNA repair [27] and TMZ resistance [106]. Although loss of ATRX expression, which is typically seen in IDH-mutant GBMs, has been linked with better survival even under consideration of IDH status [33, 34], we could not replicate these findings, perhaps due to the fact that ATRX expression was unevenly distributed (91.2% vs. 8.8%). Although 6 patients showed loss of ATRX expression, which is atypical for IDH-wildtype GBMs, these patients nevertheless demonstrated a typical course of disease and treatment and were therefore included in the patient collective.

EGFRvIII expression of 40.9% is slightly more than the mean expression reported in previous studies as mentioned above and although EGFRvIII expression showed borderline significance in Kaplan–Meier survival analysis, this did not hold up in univariate and multivariate analysis; thus, no clear statement can be made about a correlation between survival and EGFRvIII expression.

A median Ki-67 LI of 20% is in line with the malignant nature and high proliferation of GBMs [65, 66] and averages approximately in between previously published data (11 to 27% [14, 61, 62, 64]). Progression-free survival was significantly longer in patients with lower Ki-67 LI < 20%, matching previous studies [107–109]. We propose that this is a result of lower proliferation grade and therefore slower growth in tumors with lower Ki-67 LI. Furthermore, Ki-67 LI was not correlated with time to re-progression or re-recurrence, whereas retreatment was shown to have a significant impact on overall survival in our collective regardless of retreatment modality. Also, while Ki-67 LI had a significant impact on overall survival this did not hold up in multivariate analysis. This could suggest that retreatment received by our patients was sufficient to compensate for a possibly more proliferative tumor or that survival time was not long enough for initial proliferation grade to have an impact. Some studies have shown a non-significant decrease of Ki-67 LI upon recurrence [110, 111], which might account for lacking significance of overall survival in multivariate analysis; however, other studies were not able to find this decrease [108, 112]. The present study was able to show a tendency toward better OS in patients with reduction of Ki-67 LI upon progression or recurrence, with statistical significance being reached for a median Ki-67 LI quotient of 2, but with significance being missed for both an absolute median reduction of 6% as well as for Kaplan–Meier survival analysis of Ki-67 LI quotient ≤ 1 vs. > 1, which could be due to the small sample size (*n* = 24). As mentioned above, the role of Ki-67 LI as a prognostic factor is still disputed due to differing results concerning impact of Ki-67 LI on survival. A previous study has discussed suboptimal methodological approaches as a possible factor in this variance [75]. In our eyes, the strength of the present study lies firstly in the monocentric approach, seeing as Ki-67 LI detection took place at a single laboratory and with minimal examiner variance, and secondly in the inclusion of treatment criteria via analysis of the partial collective “favorable subgroup,” making the significant results regarding Ki-67 LI and PFS more reliable. Furthermore, our approach of integrating Ki-67 LI into the existing RPA score allows for inclusion of further prognostic factors and could, in our eyes, show that inclusion of Ki-67 LI can contribute to an improved assessment of survival. Concerning a possible impact of Ki-67 LI on treatment stratification, to our knowledge, no data exist regarding glioblastoma, and this was also not the focus of our study. However, Ki-67 has been shown to impact treatment response in neuroendocrine neoplasms [113], prostate cancer [114], and metastatic lung carcinoids [115], as well as impacting treatment options for breast cancer [116–118]. Such promising results in other malign entities combined with the prognostic value of Ki-67 LI as seen and discussed in our study emphasize the need for prospective trials to investigate a possible role of Ki-67 LI in stratification for treatment of glioblastoma.

MGMT promoter methylation was more common in our collective than expected, with 60% of patients having a methylated MGMT promoter compared to 40% in previously published data [12, 46, 84, 119]. A significantly better progression-free and overall survival in all collectives is in line with known data [24, 82–86], with overall survival of 13 months for non-methylated and 18 months for methylated MGMT promoter coinciding with previous studies (5.3–13 months for non-methylated and 10.3–23 months for methylated MGMT promoter [3, 83, 86]).

Regarding the modified RPA scores analyzed by us (Fig. 4), we could show that the combination of age ≥ 50 years, ECOG > 2, and biopsy reliably predicts a bad prognosis with very short survival (median PFS 4.2 months, median OS 4.3 months). On the other hand, the combination of age < 50 years, ECOG 0, and Ki-67 LI ≤ 20% might be able to identify the small collective of long-term GBM survivors irrespective of resection status, as it did in our collective. In our view, this conclusion is underscored by the fact that *p*-values of Kaplan–Meier survival analysis differentiating between score classes III and IV (RPAmod) showed lower values when this collective of long-term survivors was split off utilizing Ki-67 LI ≤ 20% (RPAki). The fact that in our collective no differentiation of survival prognosis was possible between score classes III and IV (RPAmod) or IIIa and IV (RPAki) might be either due to the fact that we did not take mental/neurological status described via working status into account as intended in the original RPA score [94] or due to the fact that age (younger vs. older) and ECOG status (lower vs. higher) even each other out concerning odd ratios.

## Conclusion

In our collective of GBM patients, a Ki-67 LI equal to or lower than 20% was an independent predictor of prolonged progression-free survival and showed significant correlation to prolonged overall survival. A methylated MGMT promoter was also an independent predictor of prolonged progression-free and overall survival. For ATRX and p53 expression, no correlation with survival could be found. For EGFRvIII expression, a borderline significant correlation was found in Kaplan–Meier survival analysis, which did not hold up to univariate or multivariate analysis.

We propose that in future, analysis of Ki-67 LI should also be included as a standard analysis and should be considered as a prognostic factor for progression-free survival upon initial diagnosis. In our opinion, further studies regarding change in or conservation of Ki-67 LI upon progression or recurrence and its influence on time to re-progression or re-recurrence and overall survival should be considered, with studies pooling collectives to reach larger patient numbers. In addition, we strongly suggest further evaluation of Ki-67 LI as part of prognosis scoring systems, as it might be able to identify long-term GBM survivors. Lastly, prospective trials to evaluate a possible impact of Ki-67 LI on treatment stratification are highly recommended.

## Supplementary Information

**Table S1:** Patient characteristics in the favorable subgroup

**Table S2:** Treatment characteristics in the favorable subgroup
