# Supplementary material for: Monocentric evaluation of Ki-67 labeling index in combination with a modified RPA score as a prognostic factor for survival in IDH-wildtype glioblastoma patients treated with radiochemotherapy
Source: Strahlenther Onkol. 2022 May 25;198(10):892–906. doi: 10.1007/s00066-022-01959-6 (PMC9515058; doi:10.1007/s00066-022-01959-6)
Supplement: Supplementary file 1 — Table S1: Patient characteristics in the favorable subgroup [file 66_2022_1959_MOESM1_ESM.docx]

**Table S1:** Patient characteristics in favorable subgroup

|  | n (%) |
| --- | --- |
| Total | 99 (100) |
| Sex  male  female | 62 (62.6)  37 (37.4) |
| ECOG at primary diagnosis  0  1  2  3  unknown | 46 (46.5)  32 (32.3)  7 (7.1)  1 (1.0)  13 (13.1) |
| Marker status  ATRX expression  yes  no  unknown  EGFRvIII overexpression  yes  no  unknown  p53 overexpression  yes  no  unknown  MGMT promoter  none-methylated  methylated  unknown  Ki-67 LI  ≤20 %  >20 %  unknown | 88 (88.9)  5 (5.1)  6 (6.1)  50 (50.5)  43 (43.4)  6 (6.1)  83 (83.8)  6 (6.1)  10 (10.1)  39 (39.4)  59 (59.6)  1 (1.0)  63 (63.6)  33 (33.3)  3 (3.0) |

*Abbreviations:* ECOG=ECOG-Status
