# Supplementary material for: Monocentric evaluation of Ki-67 labeling index in combination with a modified RPA score as a prognostic factor for survival in IDH-wildtype glioblastoma patients treated with radiochemotherapy
Source: Strahlenther Onkol. 2022 May 25;198(10):892–906. doi: 10.1007/s00066-022-01959-6 (PMC9515058; doi:10.1007/s00066-022-01959-6)
Supplement: Supplementary file 2 — Table S2: Treatment characteristics in the favorable subgroup [file 66_2022_1959_MOESM2_ESM.docx]

**Table S2:** Treatment characteristics in favorable subgroup

|  | n (%) |
| --- | --- |
| Resection status  biopsy  partial resection  complete resection | 11 (11.1)  41 (41.4)  44 (44.4) |
| Chemotherapy (TMZ/TMZ+CCNU)  yes  no | 89 (89.9)  8 (8.1) |
| Status  alive  dead | 31 (31.3)  68 (68.7) |
| Progression or recurrence  yes  no | 79 (79.8)  20 (20.2) |

*Abbreviations*: TMZ=temozolomide, CCNU=lomustine
